# Interprofessional enhanced skills training in periodontology: a qualitative study of one London pilot

**DOI:** 10.1038/bdjopen.2017.1

**Published:** 2017-02-10

**Authors:** Eloise Radcliffe, Swapnil G Ghotane, Victoria Harrison, Jennifer E Gallagher

**Affiliations:** 1 King’s College London, Faculty of Medicine and Life Sciences, Department of Primary Care and Public Health Sciences, Guy’s Campus, London, UK; 2 King’s College London Dental Institute at Guy’s, King’s College and St Thomas’s Hospitals, Division of Population and Patient Health, Denmark Hill Campus, London, UK

## Abstract

**OBJECTIVES/AIMS:**

Health Education England (HEE) London developed an innovative 2-year pilot educational and training initiative for enhancing skills in periodontology for dentists and dental hygienists/therapists in 2011. This study explores the perceptions and experiences of those involved in initiating, designing, delivering and participating in this interprofessional approach to training.

**MATERIALS AND METHODS:**

Semi-structured qualitative interviews were conducted with a purposive sample of key stakeholders including course participants (dentists and dental hygienists and/or therapists), education and training commissioners, and providers towards the end of the 2-year programme. Interviews, based on a topic guide informed by health services and policy literature, were audio-recorded and transcribed verbatim. Data were analysed based on framework methodology, using QSR NVivo 9 software to manage the data.

**RESULTS:**

Twenty-two people were interviewed. Although certain challenges were identified in designing, and teaching, a course bringing together different professional backgrounds and level of skills, the experiences of all key stakeholders were overwhelmingly positive relating to the concept. There was evidence of ‘creative interprofessional learning’, which led to ‘enhancing team working’, ‘enabling role recognition’ and ‘equipping participants for delivery of new models of care’. Recommendations emerged with regard to future training and wider health policy, and systems that will enable participants on future enhanced skills courses in periodontology to apply these skills in clinical practice.

**CONCLUSION:**

The interprofessional approach to enhanced skills training in periodontology represents an important creative innovation to build capacity within the oral health workforce. This qualitative study has provided a useful insight into the benefits and tensions of an interprofessional model of training from the perspectives of different groups of key stakeholders and suggests its application to other areas of dentistry.

## INTRODUCTION

Health Education England (HEE) London has led the way nationally in developing training for dentists, hygienists and/or therapists to provide ‘enhanced skills’ in Periodontology through a 2-year pilot programme established in 2011 at King’s College Hospital. This initiative was developed based on the concept of ‘Dentists with Special Interests’, initiated in 2004, which aimed to train practitioners working in primary care to provide supplementary services in addition to their generalist role and, therefore, addressing the ‘gap’ between primary and tertiary care levels.^1^ Guidance on implementing this concept,^2–7^ and a London pilot of training Dentists with Special Interests in Endodontics in 2009,^8^ provided the basis for HEE London to devise a programme to develop extended skills for primary dental practitioners and with the dental hygienists/therapists, an ‘interprofessional model’, as a considerable amount of periodontal care is provided by dental hygienists/therapists.

With professionals now needing to work majorly in a team setting, sentience and interaction between the professionals has become vital for delivering quality patient care,^9–11^ and thus interprofessional education is increasingly important.^12^ Defined as ‘occasions when two or more professions learn with, from and about each other, to improve collaboration and the quality of care’,^13^ it is relevant to the UK dental team, led by dentists, comprising dental hygienists and dental therapists, along with other dental care professionals (DCPs) such as dental nurses, orthodontic therapists, dental technicians and clinical dental technicians.^14,15^ Traditionally, dental hygienists and dental therapists have played a major role in delivering periodontal care under the prescription of a dentist,^16,17^ with the remit of the latter including the duties of dental hygienists, as well as dental restorations and extraction of primary (deciduous) teeth.^15,16^

Training dually qualified dental hygienist/therapists is increasingly common across the United Kingdom.^18,19^ Their roles, whether singly or dually qualified, are equivalent to mid-level dental providers, delivering basic evaluative, preventive and/or minor surgical dental care at primary care level; and are believed to have great potential to address issues of access to dental care.^18,20^ This potential received a further boost in May 2013, when General Dental Council professional policy in the United Kingdom, enabled patients to have ‘direct access’ to dental hygienists and therapists.

Improvements in oral health, with falling levels of dental caries and increasing retention of teeth which are therefore at risk of other diseases, has prompted developments in the oral healthcare systems in relation to skill mix and innovative care pathways.^21^ Epidemiological surveys highlight the prevalence of periodontal disease in the adult population, which increases with age.^22,23^ Health policy in England advocates the provision of services in a primary healthcare setting and maximising the full scope of skill mix in the dental team. Therefore, there is scope for encouraging prevention and utilising mid-level providers such as dental hygienists/therapists,^24^ and growing interest in exploring whether employing an interprofessional (skill mix) model contributes to improved patient, personnel and organisational outcomes.^25^

Considering the above policy frameworks, HEE London devised an innovative pilot skill mix, interprofessional, initiative to provide education and training for dental hygienists/therapists along with dentists. The aim was to train both the skill sets together and enable the delivery of a standard of periodontal treatment in primary dental care that is recognised by the National Health Service (NHS) commissioners/planners, teaching and regulatory organisations.^26^ Another subsidiary aim of the course was to test an interprofessional model of education for dentists and dental hygienists/therapists together. They trained together 1 day per fortnight over a 2-year period in the dental hospital.

Previously, similar schemes for Dentists with Special Interests in Periodontics and minor oral surgery have been evaluated through exploring the views of general dental practitioners and patients;^27,28^ however, there is only one study exploring the views of key stakeholders involved in development and management of such innovative programs.^8^ Therefore, the aim of this qualitative study was to explore the perceptions and experiences of all the key stakeholders regarding an interprofessional approach to training dentists and hygienists/therapists on this course.

## MATERIALS AND METHODS

Semi-structured qualitative interviews were conducted with dentists, dental hygienists and/or therapists participating on the course and other stakeholders associated directly, or indirectly, with the training initiative. Collectively, these participants will be referred to as ‘key stakeholders’. This study was approved by National Research Ethics Committee (13/NS/0102) and King’s College Hospital NHS R&D committee (King’s College Hospital 13-143). It was part of a wider mixed methods study, the overview of which is being reported as a separate paper. All of the 19 participants on the course (10 dentists and 9 with hygienist and/or therapist qualifications; the latter will collectively be referred to as hygienists/therapists hereafter) were invited to take part in an interview. Other stakeholders were purposively sampled,^29,30^ based on their role in commissioning, initiating, developing, delivering or participating in the training to ensure representation across key areas. Key stakeholders were approached by means of a letter of invitation, supported by an information sheet and consent form regarding the study, either in person or by post. Potential participants were followed up after a week either by email or phone to enquire about their decision to participate and were given an opportunity to ask questions about the study. Written consent was obtained, prior to conducting a one-to-one interview in person or over the telephone in a private office setting by one of three of the co-authors from public health backgrounds (SG, JG and VH). To ensure consistency between interviewers, a structured topic guide was used, informed by the literature. Participation was voluntary and participants were assured that all data would be anonymised. The interviews were conducted over a 6-month period (Oct 2013 to March 2014) in line with the availability of the stakeholders and course participants; interviews ranged from 30 to 60 min in duration.

Interviews were audio-recorded and transcribed verbatim. Data were analysed based on framework methodology,^31^ using QSR NVivo 9 software (supplied by QSR International Pty Ltd., Melbourne, VIC, Australia) to manage the data. The transcripts were read and re-read to enable familiarisation with the data and a coding frame was developed by one of the authors (ER) based on the initial themes and sub-themes emerging, together with the wider literature. The transcripts were coded by ER, a qualitative researcher with a background in social science, then crosschecked and verified with two other authors (JG and SG), discussing any areas of difference to ensure a consistent approach. An overarching conceptual framework was then refined to enable the data to be synthesised to examine relationships between themes and develop explanatory accounts for the data.^31^ This involved continually referring back to the original transcripts to ensure findings accurately reflected key stakeholders’ perceptions and experiences. Methods and results have been presented with reference to a comprehensive checklist for reporting qualitative research.^32^

## RESULTS

A total of 22 key stakeholders participated in interviews. Out of the 19 participants on the course invited to take part in an interview, 12 agreed to participate, comprising 4 dentists and 8 hygienists/therapists. No response was obtained from the remaining seven course participants. Out of the 27 other stakeholders invited to participate, 10 agreed. This included representatives from HEE, involved with initiating and commissioning the training course, one representative from Dental Public Health, six course educators and training providers, and one practice principal. NHS Commissioners of the training course, although invited, did not respond.

**Figure 1:**
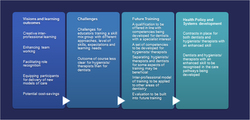
Creative interprofessional learning: key findings and recommendations.

The interprofessional aspect of the training was a very prominent theme in the data. Results are presented and discussed on the main themes identified by the key stakeholders. Challenges of the interprofessional aspect of the pilot training will then be presented and discussed. Based on the results, recommendations are discussed in relation to future training, health policy and the wider health system. Throughout this paper, results are supported by quotes from the original transcripts. Figure 1 provides a summary of the key findings and recommendations.

### Creative interprofessional learning

There was evidence of creative interprofessional learning, formal and informal, among both the dentists and DCPs. One of the visions of the course, identified by the training initiators and the educators, was to test a model of interprofessional training for qualified dentists and hygienists/therapists, as can be seen in the following quote. This model of training was regarded as innovative and therefore identified as not having been previously evaluated.

(One) aim is really to see how it would work to put dentists and hygienist/therapists, in this case, hygienists, together in the same learning environment to see how they interact and whether it’s a good model to actually train the two different skillsets at the same level. (E 201).

Interprofessional training was considered by trainers as particularly important in the field of periodontology, as hygienists/therapists are very involved in this aspect of dental care, as the quote below demonstrates.

We felt that perio is so much a skill mix topic that it was rather silly to just train the dentists without the people that would probably be doing a huge amount of the motivation, which is the hygienist. So it was very exciting to be doing it as an interprofessional project, which I still think is very exciting. And so the main thing from the end of the project is has that aim of training a skill mix been successful. (I 198).

Training initiators talked about a vision of hygienists/therapists and dentists sharing responsibility for patient care, as shown in the quote below.

If it’s perio, we don’t want to have only people who are dentists, we want the dental team involved, because health education and oral health and wellness was part of a team responsibility with the patient, rather than just the dentist, so I didn’t want ownership just to remain with the dentist but to extend it to the DCP’s (Dental Care Professionals). (I 192).

All participants reported that both groups of dentists and hygienists/therapists had benefited from the experience of interprofessional learning, describing this as ‘a two-way learning curve’ (D 206). Specifically, hygienists/therapists and dentists both reported that dentists gained skills from the hygienists/therapists on instrumentation and communication skills with patients. Hygienists/therapists and dentists both reported that hygienists/therapists gained skills from dentists on diagnosis and treatment planning. This is illustrated in the two quotes below from a hygienist/therapist and dentist.

I think it’s been a really good and positive way to learn because we’ve both learnt a lot from each other... I think a group without a mixed, a skill mix within it, the treatment on periodontology would be a bad thing because the dentists have been able to see exactly what hygienists can do for them and we’ve also been able to see and learn from them treatment planning skills and the diagnosis skills and then what we can refer onto them once we’ve completed all the non-surgical treatment. Yeah, I think everyone brings a different perspective to treatment and different experiences. (H/T 195).

I think what happened was really good, we were paired one hygienist and one dentist and I think that worked really well because, for instance, I learned hand instrumentation when I was at university, after that no. I prefer using ultrasonics all the time so that was a skill that I had kind of lost but because I was working with a hygienist and she was the one who taught me. (D 215).

The quote below illustrates the view that the course provided a valuable opportunity for dentists and hygienists/therapists to learn from each other, which did not routinely happen in dental practice due to time constraints. The setting of a dental hospital where facilities are open plan rather than closed dental surgeries may have further facilitated informal learning.

My dentist that I was working with, in the clinic, he showed me tips on injections and things and it allows them to know what we do and work better in a better relationship. So the skill mix there, I think that has to be fundamental in the training of courses like this. I think that is a really good thing that's come out of it... so (the dentists) learn communication skills from us more because that's what we do most of the time to try and get patients to clean their teeth....the dentist can learn a lot and we can learn a lot from the dentist. Because you never have that time when you can work together in close proximity to a patient and answering the different questions that that poses and them asking you ‘what do you think?’, ‘do you think?‘ which is really nice because you feel more part of the team. (H/T 205).

Educators supported these findings, stating that professional relationships developed between hygienists/therapists and dentists, and both groups benefited from shared learning.

The (interprofessional) model was amazing, and I think everybody got something out of it, the dentists and the hygienists and everybody learned from each other. (E 218).

Educators were also learning through their role in the training, as demonstrated by the following quotation, and thus it was a ‘creative’ process for them too.

I think the teachers on the course have also been impressed with the enthusiasm and actually have come away learning much more too, so I don’t think it’s been a one way process, I think it’s been a sort of multi-way process between everyone involved. (E 218).

### Enhancing team working

Another reported vision for the course was to enhance team working and communication skills to improve quality of care. Linked with this, recognising and understanding dental team member’s roles was identified as another aim of the course, as illustrated in the two quotes below.

Skill mix is part of the vision of delivering good quality dental services and in order to develop skills all the different players have to be able to work together, and I think in order to be able to work together it is desirable for them to train together, so I think training them together is actually a very positive thing because it means that everybody understands the theoretical aspect of what they’re doing. (PHC 219).

(The aim of the training was) to encourage good communication between hygienist/therapists and dentists so that they both appreciated their respected roles and realised some common paths. (E 200).

All participants spoke very positively about how well hygienists/therapists and dentists had worked together during the course. Hygienists/therapists and dentists had worked together in pairs in clinics, assisting one another reciprocally and all participants discussed how this had facilitated good team working and the development of good professional relationships. The quote below from a hygienist/therapist illustrates the way in which participants felt that professional barriers were being broken down, leading to improved teamwork that would lead to better patient care.

I think the fact that you've got dentists and hygienist/therapists working together is a really good thing... Because within the profession hygienists have a perception, and therapists have a perception about dentists, dentists have a perception about hygienist therapist... I think the working together has really helped and it is, in the future if you're working in a good team, with good interprofessional and you understand what each other... it's surely got to be better for patients. (H/T 205).

Educators’ accounts also supported participants’ reports of improved team working.

(The participants) got on fantastically well (with each other), so the whole concept of doing it I think is something that, you know, is something that should be repeated and has been a positive experience for everybody involved... we’ve got a fantastic model for, you know, building, relationships between the dental team. (E 218).

### Facilitating role recognition

Both hygienists/therapists and dentists discussed the ways in which the interprofessional approach to training had increased role recognition across the dental team and changed their wider working relationships in practice. Dentists reported that they now had much more of an understanding and appreciation of the role of hygienists/therapists after training together and the hygienists/therapists also agreed that dentists had more of an appreciation of their role and described feeling ‘valued’ (H/T 204) and given ‘respect’ (H/T 214). This point is illustrated below in quotes from a dentist and hygienists/therapist.

So dentists and hygienists/therapists providing periodontal care, I think hygienists are amazing at what they do... a lot of what I've changed in my actual clinical skills, I probably gained from my clinical partner who is a hygienist so that's been really, really good. (D 199).

I think collectively they have learned a lot from us and we have learned a lot from them. I think they have probably learned a lot and understanding our scope and our capabilities which I think is definitely lacking within the bigger market. (H/T 209).

This increased role recognition had implications for practice as some of the dentists felt their dental practice would benefit from a hygienist joining their team as they had not previously worked with one. In some cases practices were planning to employ a hygienist shortly and one had employed a hygienist already as a direct result of a dentist attending the training course, as shown in the quote below.

I have seen the benefits, I have definitely learned a lot from (the hygienists), I understand, because in dental school I wasn’t really taught about what hygienists do in their role that much, so I definitely understood their role and what skills they have... we have introduced a hygienist because we have seen the benefit of having, like from the course I have seen the benefit of having it so that is a plus point as well. And in practice having a hygienist generates revenue so obviously the practice principal is happy about that. (D 207).

These findings were supported by interview data from educators who also felt that role recognition had been an important aspect of the course for participants, as shown in the following quote.

The dentists have become much more appreciative of the skills, and often the clinical skills are vastly in excess of those of the dentist, the skills of the hygiene therapist, and so they are acknowledging the hygiene therapist much more as colleagues rather than as somebody that you go to for cleaning. (E 200).

Participants also discussed skill recognition as another outcome of the interprofessional training, which enabled them to understand their own skills and capabilities as well as those of others. This increased an understanding of skills and boundaries within the wider context of patient care, as illustrated in the quote below.

It’s really like on a football team you do not expect everybody to be doing everything. So you are all a team and you have all got one aim to win the game... no-one is more important than anybody else and you cannot function without each other. And I think the more we can integrate that sort of thing, I am sure the more cost effective care will be, I think probably the more job satisfaction everybody gets and I think ultimately more people will be treated with better outcomes... I think it has given the hygienists/therapists more confidence to go ahead and say no I am going to do that. Which I think in turn has also stopped a lot of unnecessary referrals. Because people are realising that we don‘t need to be told what to do, actually we can go ahead and do it if it’s within our expertise. (H/T 193).

### Equipping participants for delivery of new models of care

One vision of the interprofessional model discussed by the educators and training initiators was to prepare for the introduction of ‘direct acccess’, whereby patients may now be treated by dental hygienists without the supervision of a dentist, by enhancing the skills of hygienists/therapists. The concept of patients having ‘direct access’ to dental hygienist/therapists moved to a reality during this 2-year course with the support of the General Dental Council. This aim was supported by some of the hygienists/therapists who discussed preparing for ‘direct access’ as one of the reasons for enrolling on the course, as demonstrated in the quote below.

Knowing what was going on two years ago with the Dental Council and then thinking about introducing direct access for hygienists, I was pre-empting that thinking, well it would be good to do (this course) anyway but if that did come in then we would also have been trained in diagnosis, treatment planning and treating and assessing a new patient. So probably my main aim initially was pre-empting that and allowing me to complete a lot more treatment in the practice and be seen by the other, well by all the dentists in the practice to be able to do advanced treatment confidently and competently. (H/T 195).

It is currently too early to assess any impact the enhanced skills training course in periodontology may have had in relation to this particular change in the dental care system.

### Potential cost-savings

Another wider vision of the course identified by the public health consultant, training initiators and educators, related to cost-savings on the basis that it would cost the NHS less to employ hygienists/therapists to carry out routine periodontal work compared to general dental practitioners. The quote below from a training initiator frames this in terms of providing value for money for the public.

Skill mix, I think, is the right way to go. If somebody who’s a non-dentist and not trained so expensively can do some of the work of a dentist, the more routine work, and there’s evidence that they can and do it well, then I think that’s what the public will demand because it will be value for money... I think that dentistry, because we work as teams and teamwork is terribly important for patient care, then a recognition of the role of the (hygienists/therapists) is vital if dentists are going to do their job properly and if we’re going to deliver care under the new contract and the care pathways and care that is affordable and value for money. (I 198).

The above was not a view shared by dental practitioners. Furthermore, the NHS does not currently provide remuneration to dentists and hygienists/therapists for applying their enhanced skills within primary care; therefore, at present, it is too early to assess any potential cost-savings. This issue will be discussed further in the recommendations section.

### Challenges of an interprofessional model of training

Training initiators and educators discussed the challenges in relation to designing and teaching a course bringing together hygienists/therapists and dentists with different professional backgrounds and approaches. Training initiators identified some potential issues, acknowledging that dentists and hygienists/therapists have different professional backgrounds and level of skills and felt it was important that course educators take this into account, as seen in the following quote.

I would expect the teachers to appreciate the different backgrounds that (hygienists/therapists and dentists) come to the subject with and that’s the anxiety of whether that is appreciated and people understand the nuances of that. (I 198).

The public health consultant also acknowledged that it is important that the course material is appropriate for both dentists and hygienists/therapists, as seen below.

The only reservation I would have (about an interprofessional approach) would be the way in which the course material is presented so that the complexity is such that it is not too complex, so the therapist doesn’t understand, but it is not too simple that the dentist feels that they’re being patronised. So it has to be somewhere where it is suitable for both of them. (PHC 219).

In support of these potential issues identified, educators reported experiencing some challenges in teaching hygienists/therapists and dentists together.

First, challenges were identified in terms of initially designing the course for hygienists/therapists and dentists, as each group had different expectations and outcomes. From the education perspective, the overall outcome of the course for dentists was to develop skills to become dentists with enhanced skills based on the competencies for dentists with a special interest in periodontology, but the outcome for hygienists/therapists was less clear as the nature of the hygienists’ role meant they already focused in this field. However, with direct access having been recently introduced after the course began, preparing for this was highlighted as one aim for hygienists/therapists taking the course.

(The end point) is sort of clear cut for the dentists, because, you know, that’s their ultimate aim... to become, a dentist with, you know, an enhanced practitioner or whatever we’re calling them, in perio. But obviously for the hygienist, it’s, the area’s slightly less, well defined because they are, you know, by definition, if you like, already have got a special interest in perio, because that’s what they do... My understanding was that what they were, interested in really was, sort of setting themselves up for the, introduction of Direct Access. (E 218).

Second, educators suggested there were challenges teaching hygienists/therapists and dentists together because they felt the two groups had different professional knowledge and responsibilities. Three of the six educators interviewed reported experiencing specific difficulties teaching dentists and hygienists/therapists together as a group and suggested that it may have been more beneficial to separate them at certain points during the course, as can be seen in the quotes below.

When you’re trying to do a seminar and you’re trying to gear it towards dentists and hygienist/ therapists, it’s very difficult to then get the level at which you need to pitch that seminar at. Say you’re doing a seminar on (topic), you know you, it was clear from the feedback that the hygienist/therapists didn’t perhaps, they found it all a bit, oh, over my head whereas the dentists, when it was brought down the dentists found it too simple. So it was quite difficult I think on the didactic teaching sessions, to pitch at the right level to benefit both groups. (E 201).

I think it would have been a little bit more advantageous, or if the course is run again, to have some sort of separation, because I think that the applications are very different, and even if we had the same courses, called the same thing, but maybe having slightly different teaching, you could even have some lectures the same, but I think a dentist is going to be treating the patient in a very different way, often the dentist is treating the (patient) restoratively as well, so they need to be able to treatment plan in that way, and a DCP, not in the same way. What I thought we could do is run it under one umbrella, but then, and have an induction period, where you’re bringing everyone together at the same level, maybe having an intro to perio, to bring everyone, the new perio classification system, pathogenesis, those kinds of things. (E 211).

In support of the data from the educators, some participants recognised that educators experienced some challenges in providing training to an interprofessional group due to the different approaches, expectations and learning needs of the dentists and hygienists/therapists. A small number of dentists also suggested it may be beneficial to separate hygienists/therapists and dentists at some points during the course, as seen in the two quotes below. However, this was not suggested by any hygienists/therapists, who were very much in favour of being trained together with dentists. This was the only issue where the two groups of dentists and hygienists/therapists indicated differing views.

I still do think that what (hygienists/therapists) came to achieve and what dentists came to achieve was different from clinical aspects so I think that there had to be some sort separation at some points to ensure that both sets, expectations were met. (D 207).

My point is that if at the onset some courses were together and some lectures were joint lectures and then as the course went on then we could then split the hygienist and the dentist separately so that topics which are specific or which are important to the dentist, they could do it and then ones which are specific to hygienists, they could do it, and then likewise on the clinics as well, again, if it’s just a matter of bringing the patient in to just talk about oral hygiene instructions and so on and so forth then yes, maybe that clinic would be dedicated to just the hygienist. (D 212).

However, the majority of participants felt that an interprofessional approach was appropriate for an enhanced skills course in periodontology and reported very positive experiences of the teaching, as demonstrated by the following quotes.

I think that the tutors on the course found it difficult to teach a skill mix but I think it’s very doable and I think it’s actually the best way for perio to approach it because it is an area where skill mix is needed, so I think it’s a perfect way to run a course like this with DCP’s and dentists on it. (H/T 195).

The supervisors we had were excellent. And they gave more than 110 per cent... definitely with the consultants they gave us their information and they gave us their time. (H/T 204).

The findings therefore suggest that the concerns raised by educators were not reflected among the course participants.

## FUTURE RECOMMENDATIONS

### Training initiatives

This pilot study demonstrates the potential for future training programmes which, as with any new initiative, should incorporate the learning from this evaluation. It is important that the professional status of any interprofessional training course is clear and a qualification should be offered in line with competencies currently being developed for dentists with enhanced skills. This needs to be in line with NHS service requirements for periodontal care. It is recommended that a set of competencies is also developed for hygienists/therapists that will enable the outcome of interprofessional training to be clear for both dentists and hygienists/therapists.

For future training initiatives, separating hygienists/therapists and dentists for some aspects of the training may possibly be beneficial for the following indications:

- Educators found it difficult to pitch lectures at right levels to benefit both groups for topics such as gingival/periodontal surgery especially for dental hygienists/therapists.
- Dental hygienists/therapists were well versed with instrumentation regarding scaling and polishing; however, dentists felt the need of additional training.
- Treatment planning for dentists involved other restorative aspects, whereas for dental hygienists/therapists treatment planning was different with respect to their scope of practice.
- Given that the surgical module is only within the remit of dentists, that portion of the course time could have been used more helpfully for dental hygienists/therapists.

There was support from the stakeholders for the course running under one umbrella programme, whereby both groups have a mixture of common and separate learning. This recommendation is based on the interview findings from educators who experienced some challenges in training hygienists/therapists and dentists together and a small number of dentists who also felt that some separate teaching maybe beneficial in addressing the different aims and approaches of the two professional groups, as discussed.

For future interprofessional training, having hygienists/therapists and dentists from the same practice training together on a course may facilitate application of skills within a practice setting, as it may make it easier in terms of team working and implementing necessary changes. This point is illustrated by a training initiator below.

The weakness of this particular scheme from what I can see, is that we couldn’t get the dentist and their team, their hygienist and therapist all from the same practice working together, so we had different dentists from different practices, different hygienists from different practices, different therapists from different practices, so really the amount of team gelling, would probably have been less but on the positive side, it means there are more people from more practices, where they can take the messages across and try and improve the rest of the team. (I 192).

Dentists and the practice principal discussed the gap in interprofessional training in undergraduate training in general and the practice principal also felt there was a clear lack of training for dentists in managing a multidisciplinary team. Therefore, applying an interprofessional model of training in other areas of dentistry such as Paediatric dentistry maybe one way to address these gaps. It is recommended that further training courses are developed and tested based on an interprofessional model, perhaps involving staff from the same practice who can contribute to any future managed networks of clinical care.

### Health policy and systems

It was strongly recommended that funding systems are put in place to enable participants on future interprofessional training courses in enhanced skills in periodontology to apply these skills in a practice setting. There is a need for enhanced skills contracts for both dentists and dental hygienists/therapists to be established.^33–37^

The quote below from the public health consultant highlights the need for elements of the healthcare system to be reformed in order to support the application of enhanced skills in practice to improve patient care. It also highlights the need for a continuous programme of training to be established rather than a one-off course.

When we have trained these people, what is going to happen to them? We want them to deliver a specialist service in enhanced care, with enhanced skills, so how is that going to happen, where is the funding going to come from, who is going to appoint them, what is the contract going to look like, all that should have been decided before so as soon as they finish it happens.... I think the initiative is a good one, my view is that the long term impacts should have been thought through and determined, I believe that a one off training (course) doesn’t necessarily deliver the capacity that is needed to provide care for the patients, and so there should be a continuous programme, and I believe that at the end of the training, what is needed to enable these people that have been trained to provide the service should have been determined and all the systems put into place. (PHC 219).

Linked with the establishing of funding systems to support the use of enhanced skills in primary care, it is also recommended that dentists and hygienists/therapists with enhanced skills are recognised in the care pathways that are currently being developed,^38–42^ as illustrated in the following quote.

I think from the commissioners point of view as well, the move nationally is around developing care pathways, and as you know the concept of a care pathway is a journey for a patient where the patient is seen in the service most appropriate to their needs, so you want to commission a complete care pathway which starts off from, by the patient accessing a general dental practice, on the other end of the scale accessing a hospital service, and so in order to deliver that pathway you need the intermediate service, so it’s commissioner wants to commission a complete care pathway. (PHC 219).

## DISCUSSION

The interprofessional model of enhanced skills training in periodontology is the first course of its kind, and thus was conducted as a pilot within London. It is particularly relevant given the level of need for periodontal care in our ageing dentate population.^22,23^ Certain challenges were identified in designing and teaching a course bringing together hygienists/therapists and dentists with different professional backgrounds and level of skills; however, the findings suggest that this aspect of the programme was not an issue for participants. Nonetheless, the concept and experiences reported in relation to the interprofessional aspect of training were overwhelmingly positive by all groups of key stakeholders, particularly course participants. This is despite evidence suggesting that attitudinal factors among healthcare students are the most challenging barriers to successful interprofessional education.^43^ This shift towards interprofessional education is being seen globally through the World Health Organization,^44^ and the International Dental Federation.^45^

In relation to the limitations of the study, the sample may not fully represent opinions and experiences of all key stakeholders as the health service (NHS) commissioners invited to participate did not respond and not all clinicians were willing to participate. However, the sample included key stakeholders from a range of professional backgrounds, delivering both NHS and private care, from both sexes and a range of ages and ethnic backgrounds. It could be argued that interview stakeholders had a vested interest in the course and were therefore more likely to give positive feedback, particularly those who chose to participate; however, there was a range of opinions represented and the data were detailed and rich. The participants in particular were positive about the concept and principles of the scheme but were willing to provide challenge and constructive feedback about how the course should be developed and run in future.

One of the main visions of the skill mix pilot training identified by training initiators and educators was to enhance team working that was reported as a main outcome by both educators and participants. Other learning outcomes reported were interprofessional learning involving mutual role recognition, boundary setting and skill recognition that are recognised aspects of contemporary healthcare education in support of team working.^12,46–49^ The interprofessional aspect of the training was not reported by participants as one of the reasons for choosing the course; however, participants emphasised the positive working relationships developed and the breaking down of professional barriers as unexpected positive outcomes. This is an important feature of the training that should not be lost in overcoming specific challenges of teaching participants from different backgrounds.

Another vision for the pilot training identified by training initiators and educators, and a small number of hygienists/therapists, was to ‘equip participants for delivery of new models of care’, prepare for the introduction of regulatory changes that would enable patients to be treated directly by hygienists/therapists, without working under the supervision of a dentist. As the course began, the concept of ‘direct access’, for patients to dental hygienists and dental therapists, has been approved by the General Dental Council.^50,51^ As its practical implementation to healthcare has been slow, it is currently too early to assess any impact the enhanced skills training course in periodontology may have had in relation to this change in the dental care system, particularly with regard to the NHS. In the state system, additional policies need to be modified to facilitate direct access for patients, whereas private providers may begin to implement change more readily. It was clear from these findings that having a qualification would underpin the delivery of care directly by dental hygienist/therapists. Furthermore, as care pathways evolve within the NHS, harnessing the skills of practitioners with additional training will become very important to serve the population effectively,^39,52,53^ providing level 2 care, between that of a regular dentist and specialist. There is no reason why dental hygienist and/or therapists cannot form part of a delivery team within a managed care network led by one or more consultants in restorative dentistry or the mono-specialty of periodontology.

One vision for the pilot training identified by the public health consultant, training initiators and educators was potential cost-savings for the NHS; however, the NHS does not currently provide remuneration to dentists and hygienists/therapists for applying enhanced skills in periodontology within primary care. Before any future interprofessional training in enhanced skills in periodontology takes place it is strongly recommended that funding systems are reformed to enable participants to apply their skills in an NHS practice setting, including enhanced skills contracts for both dentists and hygienists/therapists. There is a need for dental contracts that recognise the role that dental professionals can play in addressing periodontal disease, and move toward rewarding outcomes in terms of a diminution in treatment need, rather than one based on the number of interventions,^54^ subject to contemporary understanding of periodontal diseases.

Although this study did not set out to test the principles of interprofessional education,^55–57^ the findings lend support particularly in relation to enabling the professions to ‘learn with, from and about each other to optimise exchange of experience and expertise, facilitating interaction, exchange and co-reflection’ to ‘compare perceptions, values, roles, responsibilities, expertise and experience’. The contribution of this pilot to ‘role recognition’ is important to consider.

This model of training should possibly be piloted for other aspects of dental care such as paediatric dentistry where dental hygiene/therapists may play a significant role.^15,21,58^ In initiating other similar training courses, it will be important to take on board the learning from this initiative. The interprofessional approach to enhanced skills training in periodontology is a creative innovation. This qualitative study has provided useful insight into the benefits and tensions of an interprofessional model of training from the perspectives of different groups of key stakeholders. The training model tested demonstrates potential and the findings from this study can enhance future programmes which should be developed to include a qualification in line with the original guidelines,^59^ and may also prove beneficial in other areas of dentistry. It is recommended that evaluation processes should be built into future training to enable longitudinal evaluation from inception to end.
